# Dietary L-citrulline modulates the growth performance, amino acid profile, and the growth hormone/insulin-like growth factor axis in broilers exposed to high temperature

**DOI:** 10.3389/fphys.2022.937443

**Published:** 2022-08-08

**Authors:** Victoria Anthony Uyanga, Jingpeng Zhao, Xiaojuan Wang, Hongchao Jiao, Okanlawon M. Onagbesan, Hai Lin

**Affiliations:** ^1^ Shandong Provincial Key Laboratory of Animal Biotechnology and Disease Control, Department of Animal Science, College of Animal Science and Veterinary Medicine, Shandong Agricultural University, Taian, China; ^2^ Department of Animal Physiology, Federal University of Agriculture, Abeokuta, Nigeria

**Keywords:** broilers, heat stress, L-citrulline, protein synthesis, growth hormone

## Abstract

Heat stress adversely affects the growth performance, muscle development, and protein metabolism in poultry. l-Citrulline (L-Cit), is a non-essential amino acid that is known to stimulate muscle protein synthesis under stress conditions. This study investigated whether L-Cit could influence the growth performance, amino acid profile, and protein metabolism in broilers exposed to high ambient temperature. In a 2 × 2 factorial arrangement, Arbor acre broilers (288 chickens) were fed with basal diet (CON) or 1% L-Cit supplemented diet and later subjected to either thermoneutral (TNZ: 24°C, 24 h/d) or heat stress (HS: 35°C for 8 h/d) environment for 21 days. The results showed that L-Cit diet promoted the body weight and body weight gain of broilers higher than the CON diet, and it further alleviated HS suppression of body weight and feed intake at certain periods (*p* < 0.05). Plasma urea, uric acid, glucose, and total cholesterol were elevated during HS, whereas, the triglyceride content was decreased (*p* < 0.05). Serum amino acids including citrulline, alanine, aspartate, and taurine were decreased by HS. L-Cit supplementation restored the citrulline level and alleviated HS induction of 3-methylhistidine (*p* < 0.05). L-Cit supplementation increased the plasma growth hormone (GH) and insulin-like growth factor-1 (IGF-1) concentration, as well as the GH concentration in the breast muscle (*p* < 0.05). The mRNA expression showed that HS elicited tissue-specific responses by upregulating some growth factors in the breast muscle, but downregulated the GH receptor, GH binding protein, and IGF-1 expression in the hypothalamus. L-Cit supplementation upregulated the GHRH and IGFBP2 expression in the hypothalamus. L-Cit also upregulated the expression of IGF-1R and IGFBP2 in the breast muscle of HS broilers. The total mTOR protein level in the breast muscle of HS broilers was also increased by L-Cit diet (*p* < 0.05). Therefore, this study demonstrated that HS negatively affected the growth performance of broilers and dysregulated the expression of growth factors related to protein metabolism. Contrarily, L-Cit promoted the growth responses of broilers *via* its stimulation of circulating GH/IGF-1 concentration. To certain extents, L-Cit supplementation elicited protective effects on the growth performance of HS broilers by diminishing protein catabolism.

## Introduction

Modern-day broiler chickens exhibit a rapid growth rate, increased muscle mass, and high feed conversion ratio (Zahoor et al., 2017). Also, these birds generate a high metabolic rate, possess low heat dissipation ability, poor heat tolerance, and are highly susceptible to changes in environmental temperature (Uyanga et al., 2022b). Heat stress is an important environmental stressor affecting poultry production. It is associated with severe detrimental effects on the growth, productivity, health, and welfare of birds (Saeed et al., 2019). Heat stress alters nutrient metabolism, which is manifested by significant changes in the concentration of blood components such as glucose, insulin, protein, total cholesterol, and triglyceride (Wang et al., 2021). The effects of heat stress on these metabolites are related to the magnitude and duration of the thermal load (Nawaz et al., 2021). Therefore, an examination of the blood biochemical profile provides succinct information on the physiological state of the body (Chand et al., 2018). Over the years, several studies have proffered measures to mitigate heat stress in poultry production. The adoption of nutritional modification including the utilization of certain amino acids has proved beneficial in alleviating heat stress effects (Kalvandi et al., 2019; Attia et al., 2020; Dionizio et al., 2021).

Amino acids (AA) are important nutrients that are involved in key physiological functions, including protein synthesis, cell turnover, tissue repair, gluconeogenesis, wound healing, and immune response (Cai et al., 2020). They act as essential precursors for various molecules and they exhibit metabolic and regulatory roles that are vital to the growth, development, health, and homeostasis of living organisms. Dietary supplementation with functional AA is beneficial in ameliorating metabolic disorders and enhancing metabolic transformations necessary for protein synthesis, and muscle growth (Wu, 2013; Uyanga et al., 2022b). Thus, AA availability is critical in maintaining protein homeostasis, especially in the skeletal muscle which is the largest reservoir of both free and peptide-bound AA (Wu, 2009).

Protein synthesis and protein breakdown are regulated through multiple pathways, such that a net increase in the rate of protein synthesis results in skeletal muscle hypertrophy (Zuo et al., 2015). The regulation of growth processes involves the complex interaction of several hormones, with the somatotropic axis playing an important role (Del Vesco et al., 2015). The growth hormone (GH) directly regulates growth responses, and a significant proportion (∼50%) of total circulating GH is bound in a complex by the growth hormone-binding proteins (Baumann, 2002). Also, insulin-like growth factor-1 (IGF-1) is an important hormone that acts downstream of GH to promote cell growth, survival, maturation, proliferation, and protein anabolism (Laron, 2001; Barclay et al., 2019). In addition, the GH/IGF-1 release is an important sensor that can induce protein synthesis *via* its activation of the mammalian target of rapamycin (mTOR) pathway and its downstream targets (Zuo et al., 2015). The mTOR signaling is a key regulator of cellular growth and proliferation that integrates signals from nutrients, growth factors, and energy status in order to regulate protein synthesis and other cellular functions (Sandri, 2008). Thus, an increase in amino acid/protein intake and the release of IGF-1 activates the mTOR complex 1 (mTORC1) pathway in the skeletal muscle (Barclay et al., 2019). However, under conditions such as heat stress, the GH and IGF-1 production becomes distorted, causing a GH-IGF uncoupling as a homeorhetic adjustment against an increased thermal load (Rhoads et al., 2010). More so, several studies have established that heat stress negatively affects the growth performance, amino acid metabolism, protein synthesis, and skeletal muscle development in broiler chickens (Ma et al., 2018; Li et al., 2021; Ma et al., 2021).

L-Citrulline is a non-essential amino acid that is found naturally in watermelon (Cynober et al., 2010). L-Citrulline is a potent precursor for arginine synthesis, an essential amino acid in poultry nutrition that is required for normal growth and development (Curis et al., 2005; Bahri et al., 2013). We had earlier demonstrated that L-Citrulline supplementation can efficiently augment arginine supply in poultry (Uyanga et al., 2020). Importantly, arginine is known to promote the overall body weight, carcass weight, lean deposition, and muscle development in poultry under normal and heat stress conditions (Fernandes et al., 2009; Castro et al., 2019; Kalvandi et al., 2022). Alongside this, it was reported that arginine can promote cell and tissue growth *via* its capacity to stimulate the release of key growth factors such as insulin, GH, and IGFs into the blood (Chromiak and Antonio, 2002; Oh et al., 2017). However, it is yet to be ascertained whether L-Citrulline can exert growth-promoting effects on poultry. L-Citrulline possesses unique properties in whole-body metabolism since it can bypass hepatic arginine catabolism to promote protein homeostasis (Moinard and Cynober, 2007). Several studies have reported on the anabolic effects of L-Citrulline during instances of citrulline and/or arginine deficiency such as malnutrition, cachexia, and intestinal impairment (Cynober et al., 2013; Ventura et al., 2013; Jourdan et al., 2015). Therefore, this study was designed to investigate the effects of L-Citrulline on the growth performance, blood biochemical indexes, amino acid profile, and the expression patterns of growth factors and somatotropic hormones in chronic heat-stressed broilers.

## Materials and methods

### Experimental animals, management, and experimental design

A total of 288, 1 day old Arbor acre broilers were obtained from Dabao Breeding Technology Co., Tai’an, China. The chicks were weighed individually (∼45 g/chick) and randomly allocated to four environmental chambers for brooding. The room temperature was set at 32°C for the first 7 days, then it was gradually adjusted to reach 24°C at 21 days of age. Each of the environmental chambers had double-tiered battery cages units, with a total of 24 cage partitions. The cage dimension was 38 × 47 × 35 cm. Each cage partition housed three birds, and three partitions (9 birds) constituted 1 replicate of the treatment.

Basal diet was fed from day-old to establish uniform startup of the flock. Experimental birds were fed two dietary treatments, either basal diet (CON) or basal diet supplemented with 1% L-Citrulline (L-Cit) from 7 days old until 42 days of age (Table 1). L-Citrulline was bought from Shandong Fosun Biotechnology Co., Ltd., China, and the diets were formulated to meet or exceed NRC recommendations (NRC, 1994). At 22 days old, the broilers were exposed to two environmental conditions until 42 days of age. The temperature of two environmental chambers was adjusted to high temperature of 35°C for 8 h/d (HS), then returned to 24°C for the remaining 16 h/d to mimic cyclic heat stress condition. Another two environmental chambers were maintained at thermoneutral condition (TNZ) of 24°C for 24 h/d. The experiment was arranged as a 2 × 2 factorial design having environment (TNZ vs. HS) as the first factor, and diet (CON vs. L-Cit) as the second factor. The experimental groups included 4 treatments, 8 replicates, and 9 chickens per replicate, consisting of birds housed at TNZ and fed CON diet (TNZ + CON); birds housed at TNZ and fed L-Cit diet (TNZ + L-Cit); birds housed at HS and fed CON diet (HS + CON), and birds housed at HS and fed L-Cit diet (HS + L-Cit). Broilers had *ad libitum* access to feed and water during the 6 weeks trial. Data were collected for feed intake and body weight on weekly basis.

**TABLE 1:** Composition and nutrient levels of experimental diets (as-fed basis) %.

Ingredients (%)	d1-21		d22-42	
	CON	L-Cit	CON	L-Cit
Corn (8.5% CP)	55.02	55.02	59.61	59.61
Soybean meal (43% CP)	37.02	37.02	31.71	31.71
Soybean oil	3.87	3.87	4.73	4.73
Limestone	1.19	1.19	1.27	1.27
Dicalcium phosphate	1.68	1.68	1.63	1.63
NaCl	0.30	0.30	0.28	0.28
L-Lys·HCl (99%)	0.20	0.20	0.18	0.18
DL-Met (99%)	0.21	0.21	0.14	0.14
Choline Chloride (50%)	0.26	0.26	0.20	0.20
Vitamin premix^a^	0.05	0.05	0.05	0.05
Mineral premix^b^	0.20	0.20	0.20	0.20
l-Citrulline	—	1.00	—	1.00
Calculated Nutrient level				
CP, %	21.00	21.00	19.00	19.00
ME, kcal/kg	3,000	3,000	3,100	3,100
Ca, %	0.90	0.90	0.90	0.90
Available P	0.45	0.45	0.43	0.43
Lys	1.23	1.23	1.10	1.10
Met	0.54	0.54	0.45	0.45
Met + Cys	0.89	0.89	0.77	0.77
Thr	0.86	0.86	0.78	0.78

a Vitamin premix provided the following per kilogram of diet: VA (retinyl acetate) 10 000 IU, VD_3_ (cholecalciferol) 2,000 IU, VE (DL-α-tocopheryl acetate) 11.0 IU, VK, 1.0, VB_1_ 1.2, VB_2_ 5.8, VB_6_ 2.6, VB_12_ 0.012, niacin 66.0 mg, pantothenic acid (calcium pantothenate) 10.0, biotin 0.20, folic acid 0.70 mg.

b Mineral premix provided the following per kilogram of diet: Mg 100, Zn 75, Fe 80, I 0.65, Cu 8.0, Se 0.35 mg.

### Blood collection and tissue sampling

At 42 days old, 8 birds per treatment were selected for blood sampling. Blood was collected from the wing vein into anticoagulated tubes and coagulated tubes, then centrifuged at 4°C, 1,500 *g* for 10 min to obtain plasma and serum samples respectively. Blood samples were stored at −20°C until analysis. The broilers were sacrificed by decapitation and exsanguination. Tissue samples including the breast muscle, liver, kidney, heart, spleen, thymus, and bursa were isolated and weighed. After decapitation, the hypothalamus was dissected from the brain using surgical scissors and mini tweezers. The landmarks were rostral of the optic chiasm and caudal to the mammillary bodies for each chicken brain as previously reported (Xu et al., 2011). The sampled tissues were snap-frozen in liquid nitrogen, then stored at −80°C for molecular analysis.

### Production performance of broilers

The body weight and feed intake of broilers were weighed every week during the experiment. The average body weight, body weight gain, average feed intake, and feed conversion ratio were calculated weekly throughout the experimental period.

### Determination of protein content

Total protein was extracted from breast muscle tissues in the proportion of 1: 9 tissue to saline volume. The tissue was homogenized at 4°C, 12,000 rpm for 10 min, and the supernatant was collected. The Bicinchoninic acid (BCA) Kit (NCM Biotech, China) was used for protein determination. Briefly, 20 ul of each sample was added to the 96-well plate and the standard working solution was used to prepare the standard curve. The reaction mix was prepared in a volume of 50: 1 (Reagent A: Reagent B), and a 200µL volume was added to each well. Plates were incubated at 37°C for 15 min and absorbance was read at 570 nm. The reaction absorbance was read using a microplate reader (Elx808, Bio-Tek Winooski, VT, United States).

### Determination of plasma metabolites and hormonal assays

Plasma metabolites including alanine transaminase, aspartate aminotransferase, urea, uric acid, glucose, triglycerides, and total cholesterol were measured using an automatic biochemical analyzer (Hitachi L-7020, Hitachi High-Technologies Corp., Tokyo, Japan). The growth hormone (GH) and insulin-like growth factor 1 (IGF-1) concentration were analyzed using commercial enzyme-linked immunosorbent assay kits according to manufacturer’s guidelines (Jiancheng Bioengineering Institute, Nanjing, Jiangsu, China).

The assay principle involved a double antibody sandwich method to determine the level of chicken GH or IGF-1 in the sample. A purified chicken GH or IGF-1 antibody-coated 96 microtiter plate well was used to form a solid-phase antibody. The detected antibodies were labeled with horseradish peroxidase (HRP) to form an antibody-antigen-enzyme-antibody complex. After washing, the addition of 3,3′, 5,5″-tetramethylbenzidine (TMB) substrate solution gave off a blue colored reaction. The reaction was terminated with the addition of sulfuric acid solution to produce a yellow color and the concentration of each sample was then determined by comparing the optical density of the sample to that of the standard curve. The absorbance was determined at 540 nm (Elx808, Bio-Tek Winooski, VT, United States).

### Determination of serum amino acids

To detect the serum-free AA, 40 mg salicylic acid was added to 1ml serum for deproteinization. The samples were vortexed (Guohua Electric Appliance, China), and stored at 4°C overnight. Samples were later centrifuged at 4°C, 12,000 rpm for 30 min s. The supernatant was collected and filtered (0.22 μm), then the absorbance of individual samples was determined by ion-exchange chromatography using Hitachi L-8900 Amino acid Analyzer (HITACHI High-Tech Science, Japan) under physiological fluid analysis conditions.

### Real-time PCR analyses

Total RNA from the hypothalamus and breast muscle was isolated using the NcmZol reagent (NCM Biotech, China), according to the manufacturer’s instructions. The RNA quality and concentration were determined, and the absorbance ratio of A260/280 nm ranged from 1.80 to 2.00 (DS-11 spectrophotometer, Denovix Incorporated, United States). RNA was transcribed into complementary DNA (cDNA) using the HiFiScript cDNA synthesis kit (CWBIO, China) in a 20 μl final reaction volume as previously described (Uyanga et al., 2022a). The reaction was carried out using the Genemate T960 Touch thermocycler (Heal Force Bio-Meditech Holdings Limited, China). The cDNA was diluted using a 5-fold dilution with DNase/RNase-Free water. A 20 µl PCR reaction mix was prepared which consisted of 2 µl cDNA template, 0.4 µl each of forward and reverse primers, 10 µl of 2x MagicSYBR mixture, 0.2 µl ROX reference dye, and 7 µl ddH_2_O (CWBIOtech, China). Specific primer pairs for selected genes (Table 2) were designed using NCBI Primer-BLAST program and Beacon Designer 8 software based on published target sequences (Jia et al., 2018). Primers were synthesized by Sangon Biotechnology (Shanghai, China) as shown in Table 2. A standard curve and a melt curve were determined to calculate the efficiency of the real-time PCR primers (Kang et al., 2017; Madkour et al., 2021). Real-time qRT-PCR was performed on ABI QuantStudio five Real-Time PCR Instrument (Applied Biosystems, ThermoFisher Scientific, United States). Thermal cycling was initiated with an initial denaturation stage of 30 s at 95°C, and this was followed by 40 cycles of denaturation for 5 s at 95°C, annealing and extension for 30 s at 60°C and melt curve analysis (15 s at 95°C; 1 min s at 60°C; 15 s at 95°C, and 30 s at 50°C). Primers were normalized against the mRNA level of β-actin as housekeeping gene and the TNZ + CON group was used as calibrator. The relative expression of the target genes were analyzed using the 2^−ΔΔCT^ method (Livak and Schmittgen, 2001).

**TABLE 2:** List of primers used for real-time PCR analysis.

Gene	Primer sequence (5′to 3′)	Orientation	^a^Accession No	Product length	Annealing Temperature(^o^C)
GHR	CAA​GGT​GGG​AAG​AGC​ACA​GT	Forward	M74057.1	231	58
	TCC​ATA​CTT​GGG​GTT​TCT​GC	Reverse			
GHRH	AGT​CAC​AAG​CTC​CAT​CTC​CTC​TCC	Forward	XM_015296360	299	58
	CTG​GGC​TGC​TCT​CAC​TGT​TTC​TG	Reverse			
GHBP	GGC​ACT​GGT​CTG​TGC​AAA​T	Forward	DQ138367.1	192	60
	TCC​GGA​CAT​TCT​TTC​CAG​TC	Reverse			
IGF-1	AGA​CGA​GGC​TTC​TAC​TTC​A	Forward	NM_001004384.2	152	54
	GCAGATTTAGGTGGCTTT	Reverse			
IGF1R	CAG​GAA​CGA​TGG​AGG​AGA​AG	Forward	AF041800.1	199	58
	ACG​CAA​GCA​GTG​TTG​TTG​TC	Reverse			
IGFBP2	CAC​AAC​CAC​GAG​GAC​TCA​AA	Forward	NM_205359.1	205	58
	CAT​TCA​CCG​ACA​TCT​TGC​AC	Reverse			
β-Actin	TGC​GTG​ACA​TCA​AGG​AGA​AG	Forward	NM_205518	300	58
	TGC​CAG​GGT​ACA​TTG​TGG​TA	Reverse			

a Accession number refers to Gene bank (NCBI). GH, receptor (GHR); GH, releasing hormone (GHRH); GH-binding protein (GHBP); IGF-1, Insulin-like growth factor-1 (IGF-1); IGF-1, receptor (IGF-1R); IGF1-binding proteins 2 (IGFBP2).

### Immunoblotting analyses

Total protein extract from breast muscle was prepared by homogenizing in RIPA lysis buffer (Beyotime, China), proteolytic protease and phosphatase inhibitor cocktail (NCM Biotech, China). Protein concentration was measured using BCA protein assay kit (NCM Biotech, China). Total protein (60 µg) was electrophoresed by SDS-PAGE and transferred to PVDF membranes (Millipore, Germany) using a transfer apparatus (Bio-Rad, United States). The membranes were blocked with blocking buffer (Beyotime, China) at room temperature for 1 h and then incubated with primary antibodies against the target protein. The primary antibodies used were: anti-phospho-4E-BP1 (Thr 37/46), anti-4E-BP1, anti-phospho-AKT, anti-AKT, anti-phospho-mTOR (Ser 2,448), anti-mTOR (Cell Signaling Technologies, Danvers, MA, United States), and anti-tubulin (Beyotime, China). The primary antibodies were incubated overnight at 4°C, thereafter, membranes were incubated with corresponding secondary antibodies [horseradish peroxidase-linked goat anti-rabbit or mouse IgG (Beyotime China)] at 4°C for 6–8 h. Enhanced chemiluminescence [ECL Plus A and B (Beyotime, China)] was used to detect the protein–antibody complexes, and the densitometric analysis of western blot bands was performed using the Fusion FX software (Vilber, France). Tubulin was used as an internal control to represent the relative abundance of each target protein.

### Statistical analysis

Data were analyzed using two-way ANOVA, having environment (TNZ vs. HS) and diet (CON vs. L-Cit) as the main effects. Average feed intake and feed conversion ratio at 1–21 days were analyzed using One way ANOVA. Feed intake, body weight, and body weight gain from 22 to 42 days were analyzed using two-way ANOVA to evaluate the main effect of environment, diet, and environment × diet interaction. The data were expressed as mean ± SEM and analysis was done using Statistical Analysis Software (SAS version 8.1; SAS Institute Inc., Cary, NC, United States). Duncan’s Multiple Range Test was used to analyze mean comparisons. Charts were designed using GraphPad Prism, version 8.0.2, (GraphPad Software Inc., La Jolla, California, USA). Differences were considered significant at *p* < 0.05.

## Results

### Production performance and organ index of broilers

Table 3 shows the production performance of broilers during the trial. L-Cit supplementation significantly increased the body weight of broilers from wk 3 to wk 6 of the experiment than the CON diet (*p* < 0.05). Under HS exposure, the body weight was significantly lowered compared to the TNZ birds from wk 4 to wk 6 of the experiment (*p* < 0.05). Also, there was a significant effect of environment × diet interaction at wk 6 of the experiment (*p* < 0.05). It was observed that the HS + CON broilers had the lowest body weight compared to the TNZ + CON, TNZ + L-Cit, and HS + L-Cit groups. This revealed that during HS, L-Cit supplementation restored the body weight of broilers similar to TNZ condition.

**TABLE 3:** Effects of heat stress and L-citrulline supplementation on the production performance of broilers.

Parameters	Experimental groups				*p* Value		
	TNZ + CON	TNZ + L-Cit	HS + CON	HS + L-Cit	Envt	Diet	Envt*Diet
Body weight(g/bird)							
D0	45.43 ± 0.17	45.60 ± 0.10				0.409	
wk1	158.47 ± 2.24	162.42 ± 2.26				0.225	
wk2	398.71 ± 8.43	417.22 ± 11.28				0.199	
wk3	733.96 ± 12.93^y^	785.95 ± 13.34^x^				0.009	
wk4	1,211.13 ± 18.53	1,276.39 ± 40.76	1,077.34 ± 19.48	1,217.04 ± 39.50	0.005	0.003	0.246
wk5	1721.98 ± 25.92	1825.16 ± 48.19	1,547.27 ± 37.82	1742.59 ± 57.69	0.007	0.002	0.304
wk6	2,311.76 ± 45.57^a^	2,351.58 ± 49.63^a^	1971.88 ± 64.39^b^	2,283.39 ± 95.78^a^	0.004	0.011	0.041
Body weight gain (g/bird)							
wk1	113.04 ± 2.31	116.82 ± 2.25				0.251	
wk2	240.24 ± 8.01	254.80 ± 10.57				0.281	
wk3	335.25 ± 10.52^y^	368.73 ± 4.25^x^				0.006	
wk4	452.72 ± 22.80	494.91 ± 24.11	367.83 ± 22.79	426.62 ± 27.76	0.004	0.048	0.737
wk5	510.85 ± 8.36	548.77 ± 10.66	469.93 ± 23.80	525.56 ± 25.44	0.097	0.019	0.639
wk6	566.64 ± 33.46^a^	501.76 ± 16.91 ^ab^	433.92 ± 46.43^b^	506.24 ± 18.05 ^ab^	0.054	0.906	0.041
Feed intake (g/bird)							
wk1	135.11 ± 2.47	139.34 ± 2.98				0.293	
wk2	426.92 ± 20.57^x^	328.44 ± 19.24^y^				0.004	
wk3	476.95 ± 11.71	481.99 ± 18.87				0.823	
wk4	680.39 ± 15.56^b^	716.59 ± 39.47^b^	570.49 ± 19.99^c^	793.17 ± 17.32^a^	0.511	<.0001	0.001
wk5	994.83 ± 28.62	1,012.35 ± 58.18	860.27 ± 25.24	956.73 ± 47.77	0.032	0.188	0.358
wk6	1,281.82 ± 130.83	1,099.93 ± 17.38	969.79 ± 30.00	984.29 ± 62.48	0.033	0.379	0.304
Feed conversion ratio							
d1-21	1.47 ± 0.05^x^	1.30 ± 0.04^y^				0.027	
d22-42	1.92 ± 0.09	1.84 ± 0.04	1.90 ± 0.09	1.88 ± 0.05	0.876	0.492	0.705

Data were presented as mean ± SEM (*n* = 8); ^x,y^ Different letters indicate significant main effect of diet at *p* < 0.05; ^a,b,c^ Different letters indicate significant differences in environment × diet interaction at *p* < 0.05. TNZ + CON = thermoneutral + control diet; TNZ+ L-Cit = thermoneutral + 1% l-citrulline diet; HS + CON = heat stress + control diet; HS + L-Cit = heat stress + 1% l-citrulline diet.

Similarly, the body weight gain was higher in broilers fed L-Cit compared to the CON diet from wk 3 to wk 5 of the experiment (*p* < 0.05). However, HS significantly reduced the body weight gain at wk 4 of the experiment (*p* < 0.05). In addition, there was a significant environment × diet interaction at wk 6 of the experiment, which showed that the HS + CON broilers had decreased body weight gain compared to the TNZ + CON group (*p* < 0.05). An assessment of the feed intake showed that at wk 2 of the experiment, broilers fed L-Cit diet had lesser feed intake than the CON birds, however, this was reversed at wk 4 of the experiment. Moreso, HS exposure decreased the feed intake of broilers at wk 5 and wk 6 of the experiment (*p* < 0.05). There was also a significant environment × diet interaction at the wk 4 of the experiment, which showed that the HS + L-Cit broilers had the highest feed intake, whereas, the HS + CON broilers had the lowest feed intake (*p* < 0.05). In addition, the feed conversion ratio of broilers from d1-21 of the experiment was decreased by L-Cit diet compared to the CON diet, however, the feed conversion ratio did not differ among treatments at d22-42 of the experiment.

Table 4 shows that the absolute weights of the heart, spleen, thymus, and bursa were unaffected by treatments (*p* > 0.05). Likewise, the relative weights of the liver, heart, spleen, thymus, and bursa were unchanged (*p* > 0.05). However, it was observed that HS decreased the absolute and relative weight of the kidney compared to the TNZ group. Also, the absolute weight of the liver and bursa was decreased in broilers exposed to HS condition (*p* < 0.05).

**TABLE 4:** Effects of heat stress and L-citrulline supplementation on organ indexes of broiler chickens.

Parameters	Experimental groups				*p* Value		
	TNZ + CON	TNZ + L-Cit	HS + CON	HS+ L-Cit	Envt	Diet	Envt. * Diet
Absolute organ weights (g)							
Liver	36.26 ± 1.43	32.38 ± 2.85	37.36 ± 1.96	31.64 ± 2.14	0.034	0.934	0.673
Kidney	5.93 ± 0.12	4.98 ± 0.50	6.30 ± 0.19	5.19 ± 0.35	0.004	0.378	0.806
Heart	7.34 ± 0.62	6.64 ± 0.84	7.48 ± 0.32	6.51 ± 0.42	0.168	0.992	0.825
Spleen	1.93 ± 0.16	1.70 ± 0.30	2.03 ± 0.16	1.88 ± 0.42	0.512	0.63	0.895
Thymus	5.11 ± 0.51	4.83 ± 1.21	5.21 ± 0.35	4.59 ± 1.17	0.616	0.94	0.852
Bursa	3.80 ± 0.38	2.20 ± 0.39	3.14 ± 0.25	2.78 ± 0.49	0.017	0.911	0.121
Relative organ weights (%)							
Liver	1.70 ± 0.04	1.70 ± 0.06	1.64 ± 0.06	1.57 ± 0.07	0.565	0.109	0.584
Kidney	0.28 ± 0.01	0.26 ± 0.01	0.28 ± 0.01	0.26 ± 0.01	0.02	0.819	0.944
Heart	0.34 ± 0.02	0.35 ± 0.03	0.33 ± 0.01	0.32 ± 0.01	0.937	0.359	0.774
Spleen	0.09 ± 0.01	0.09 ± 0.01	0.09 ± 0.01	0.09 ± 0.02	0.83	0.966	0.875
Thymus	0.09 ± 0.01	0.09 ± 0.01	0.09 ± 0.01	0.09 ± 0.02	0.787	0.739	0.909
Bursa	0.18 ± 0.02	0.12 ± 0.02	0.14 ± 0.01	0.13 ± 0.02	0.06	0.519	0.131

Data were presented as mean ± SEM, with significant difference at *p* < 0.05 (*n* = 8). TNZ + CON = thermoneutral + control diet; TNZ+ L-Cit = thermoneutral + 1% l-citrulline diet; HS + CON = heat stress + control diet; HS + L-Cit = heat stress + 1% L-citrulline diet.

### Plasma metabolites

Table 5 shows that HS exposure increased the plasma urea, uric acid, glucose, and total cholesterol, but it decreased the triglyceride content compared to the TNZ condition (*p* < 0.05). HS did not affect the alanine transaminase and aspartate aminotransferase contents (*p* > 0.05). In addition, there was no significant main effect of diet (CON vs. L-Cit) or environment × diet interaction on the alanine transaminase, aspartate aminotransferase, urea, uric acid, glucose, total cholesterol, and triglyceride contents (*p* > 0.05).

**TABLE 5:** Effects of L-citrulline supplementation on the plasma metabolites of broilers during heat stress.

Parameters	Experimental groups				*p* Value		
	TNZ+ CON	TNZ + L-Cit	HS+ CON	HS + L-Cit	Envt	Diet	Envt x Diet
Alanine transaminase (U/L)	17.88 ± 1.33	22.38 ± 2.51	20.75 ± 1.81	20.13 ± 2.34	0.880	0.353	0.222
Aspartate aminotransferase (U/L)	237.00 ± 12.69	310.88 ± 31.44	292.38 ± 25.10	315.13 ± 36.07	0.292	0.093	0.365
Urea (mmol/L)	0.15 ± 0.04	0.27 ± 0.05	0.45 ± 0.16	0.89 ± 0.38	0.035	0.186	0.463
Uric acid (µmol/L)	141.75 ± 17.92	146.13 ± 13.01	288.50 ± 73.69	283.13 ± 96.15	0.029	0.994	0.938
Glucose (mmol/L)	11.58 ± 0.30	11.77 ± 0.37	12.28 ± 0.46	12.85 ± 0.39	0.028	0.334	0.632
Triglyceride (mmol/L)	0.27 ± 0.02	0.26 ± 0.03	0.21 ± 0.03	0.17 ± 0.01	0.001	0.269	0.568
Total cholesterol (mmol/L)	2.58 ± 0.16	2.77 ± 0.17	3.40 ± 0.25	3.68 ± 0.41	0.003	0.382	0.869

Data were presented as mean ± SEM, with significant difference at *p* < 0.05 (*n* = 8). TNZ + CON = thermoneutral + control diet; TNZ + L-Cit = thermoneutral + 1% l-citrulline diet; HS + CON = heat stress + control diet; HS + L-Cit = heat stress + 1% l-citrulline diet.

### Serum amino acid profile

Table 6 shows that the concentration of several essential AA including methionine, histidine, phenylalanine, arginine, isoleucine, leucine, threonine, valine, and glycine were not significantly affected by environment, diet, or environment × diet interaction (*p* > 0.05), except for lysine. It was observed that the serum lysine content was decreased with L-Cit supplementation compared to the broilers fed CON diet (*p* < 0.05). Among the non-essential AA, HS significantly decreased the contents of citrulline, alanine, aspartate, taurine, and 3methylhistidine compared to the TNZ condition (*p* < 0.05). In addition, L-Cit supplementation lowered the tyrosine and 3methylhistidine contents compared to the CON diet (*p* < 0.05).

**TABLE 6:** Effects of heat stress and L-citrulline supplementation on the serum amino acid composition of broilers.

Amino acids (µg/ml)	Experimental groups				*p* Value		
	TNZ + CON	TNZ + L-Cit	HS + CON	HS + L-Cit	Envt	Diet	Envt * Diet
Essential AA							
Methionine	7.47 ± 0.48	7.87 ± 1.04	7.13 ± 0.56	6.48 ± 0.56	0.236	0.863	0.467
Lysine	2.65 ± 0.15	2.58 ± 0.19	3.36 ± 0.38	2.28 ± 0.24	0.444	0.041	0.071
Histidine	8.85 ± 0.54	9.22 ± 1.01	9.94 ± 1.01	7.31 ± 0.37	0.611	0.171	0.072
Phenylalanine	28.64 ± 1.95	27.42 ± 1.95	23.69 ± 2.38	29.33 ± 2.15	0.480	0.309	0.119
Arginine	56.94 ± 2.74	50.87 ± 6.03	62.40 ± 6.51	58.17 ± 3.99	0.230	0.330	0.861
Isoleucine	4.52 ± 0.33	5.85 ± 1.09	4.32 ± 0.51	3.90 ± 0.47	0.131	0.512	0.216
Leucine	12.07 ± 0.86	11.46 ± 0.69	13.85 ± 1.56	10.84 ± 0.75	0.585	0.094	0.259
Threonine	54.35 ± 6.25	44.58 ± 6.17	39.18 ± 5.17	39.54 ± 5.64	0.094	0.425	0.392
Valine	12.40 ± 1.13	11.98 ± 0.92	11.18 ± 1.57	10.57 ± 1.12	0.292	0.676	0.939
Cysteine	44.30 ± 3.51	39.47 ± 3.47	44.64 ± 5.43	41.46 ± 3.82	0.783	0.347	0.845
Glycine	26.24 ± 1.36	23.27 ± 2.49	22.20 ± 1.10	23.10 ± 1.28	0.220	0.544	0.259
Non-essential AA and Peptides							
Citrulline	123.88 ± 7.40^a^	115.31 ± 2.66^a^	95.02 ± 6.25^b^ ^b^	116.47 ± 7.70^a^	0.046	0.337	0.032
Ornithine	59.17 ± 7.51	54.93 ± 10.20	49.30 ± 6.80	47.17 ± 8.10	0.299	0.705	0.900
Serine	75.64 ± 3.74^a^	56.78 ± 7.92^b^	51.98 ± 4.48^b^	65.42 ± 5.77^ab^	0.209	0.646	0.010
Alanine	70.62 ± 5.73	60.20 ± 8.69	50.54 ± 4.20	54.06 ± 5.40	0.047	0.587	0.277
Aspartate	9.67 ± 1.00	10.41 ± 0.77	7.11 ± 0.92	8.44 ± 0.87	0.017	0.255	0.743
Taurine	68.39 ± 9.99	50.22 ± 5.48	35.77 ± 5.13	42.41 ± 7.12	0.007	0.413	0.085
Cystathionine	6.55 ± 0.44	6.19 ± 0.77	5.84 ± 0.64	4.89 ± 0.47	0.108	0.291	0.630
Tyrosine	28.35 ± 1.72	23.76 ± 1.62	30.29 ± 2.56	25.81 ± 1.07	0.287	0.020	0.977
3Methylhistidine	3.23 ± 0.34^b^	3.30 ± 0.20^b^	11.52 ± 1.96^a^	2.65 ± 0.29^b^	0.0001	<.0001	<.0001
Anserine	7.87 ± 0.61^ab^	8.86 ± 1.03^ab^	10.29 ± 1.13^a^	7.06 ± 0.77^b^	0.740	0.235	0.030
Carnosine	5.35 ± 0.25^b^	9.66 ± 2.00^a^	6.95 ± 0.70^ab^	5.78 ± 0.39^b^	0.319	0.174	0.021
Essential AA	232.19 ± 12.97	210.98 ± 12.82	219.68 ± 8.79	209.89 ± 11.46	0.562	0.192	0.626
Non-essential AA	475.19 ± 29.47	440.36 ± 20.56	373.92 ± 14.14	399.76 ± 28.76	0.007	0.853	0.217
Total AA	707.38 ± 41.69	661.73 ± 27.37	593.60 ± 18.76	609.65 ± 38.00	0.017	0.653	0.351

Data were presented as mean ± SEM (*n* = 8).

TNZ + CON = thermoneutral + control diet; TNZ + L-Cit = thermoneutral + 1% l-citrulline diet; HS + CON = heat stress + control diet; HS + L-Cit = heat stress + 1% l-citrulline diet; ^a,b^ Different letters indicate significant differences in environment × diet interaction at *p* < 0.05.

There was a significant interaction between environment and diet on the contents of some non-essential AA and peptides (*p* < 0.05). The citrulline content was decreased by HS + CON compared to the TNZ + CON, TNZ + L-Cit, and HS + L-Cit groups (*p* < 0.05). Serine was decreased in the TNZ + L-Cit, and HS + L-Cit groups compared to the TNZ + CON group (*p* < 0.05). It was also observed that HS + CON significantly elevated the 3-methylhistidine content higher than the other treatments of TNZ + CON, TNZ + L-Cit, and HS + L-Cit.

The serum concentration of peptides showed that HS + CON increased the anserine content higher than the HS + L-Cit group (*p* < 0.05). Also, the carnosine content was increased by TNZ + L-Cit compared to the TNZ + CON and HS + L-Cit groups (*p* < 0.05).

### Hypothalamic expression of somatotropic axis-related genes

**FIGURE 1:**
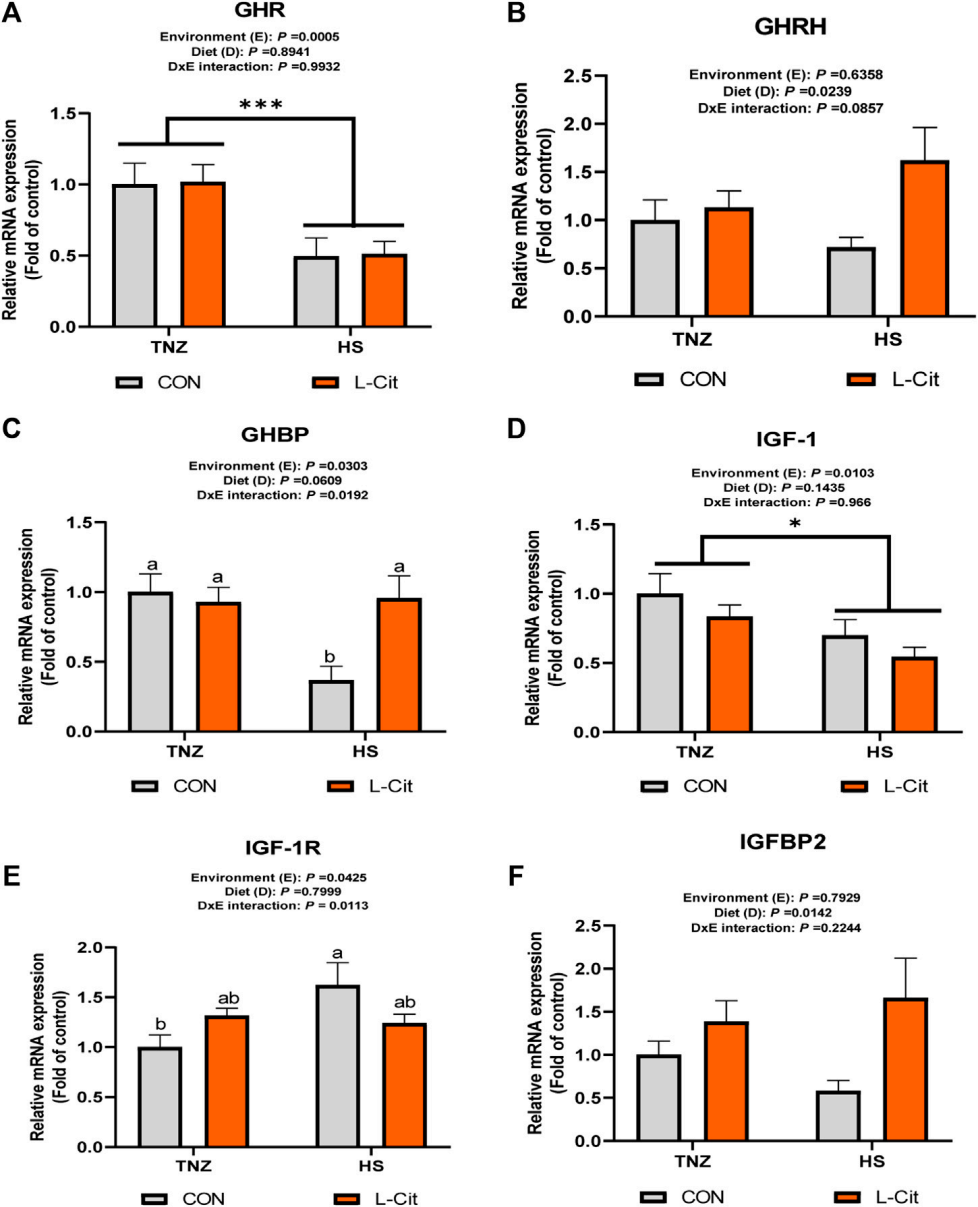
Effects of heat stress and l-citrulline supplementation on somatotropic axis gene in the hypothalamus of broilers **(A**) Growth hormone receptor (GHR) **(B)** Growth hormone releasing hormone (GHRH) **(C)** Growth hormone binding protein (GHBP) **(D)** Insulin-like growth factor-1 (IGF-1) **(E)** Insulin-like growth factor-1 receptor (IGF-1R) **(F)** Insulin-like growth factor-1 binding protein 2 (IGFBP2). Data were presented as mean ± SEM (*n* = 8). Means with different alphabetical superscripts indicate significant differences in environment × diet interaction at *p* < 0.05; Asterisk indicate significant effect of environment at *p < 0.05; ***p < 0.001.

Figure 1 shows the mRNA expression of growth factors in the hypothalamus of broilers. The hypothalamic expression of GHR was downregulated by HS compared to the TNZ condition (Figure 1A; *p* < 0.05). The GHRH expression was upregulated by L-Cit diet compared to the CON diet (Figure 1B; *p* < 0.05), whereas, GHBP expression was significantly downregulated by HS exposure (Figure 1C; *p* < 0.05). Also, there was a significant environment × diet interaction, such that the HS + CON group had downregulated GHBP expression compared to other treatments (Figure 1C; *p* < 0.05). The hypothalamic IGF-1 expression was also downregulated during HS condition compared to the TNZ group (Figure 1D; *p* < 0.05). In contrast, HS upregulated the hypothalamic expression of IGF-1R (Figure 1E; *p* < 0.05). There was an interaction between environment and diet such that the HS + CON group upregulated the mRNA expression of IGF-1R compared to the TNZ + CON group (Figure 1E; *p* < 0.05). In addition, the hypothalamic expression of IGFBP2 was upregulated by L-Cit compared to the CON diet (Figure 1F; *p* < 0.05).

### Concentration of somatotropic hormones in the plasma and breast muscle tissue

**FIGURE 2:**
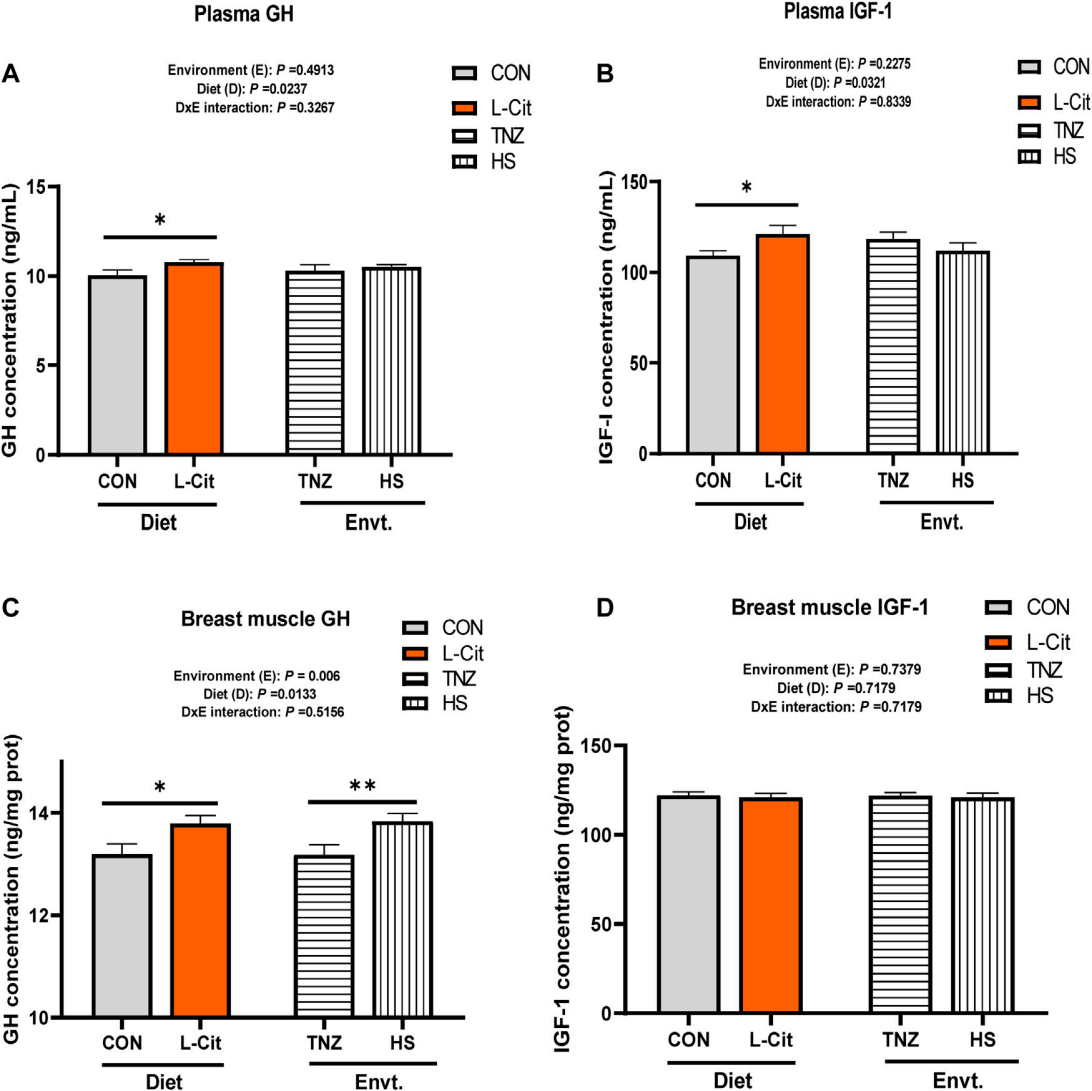
Effects of heat stress and l-citrulline supplementation on the concentration of somatotropic axis hormones **(A)** Plasma Growth hormone **(B)** Plasma Insulin-like growth factor-1 **(C)** Growth hormone in breast muscle **(D)** Insulin-like growth factor-1 in breast muscle. Data were presented as mean ± SEM (*n* = 8). Asterisk indicate significant main effect at *p < 0.05; **p < 0.01

The plasma GH concentration was significantly increased by L-Cit supplementation compared to the CON diet (Figure 2A; *p* < 0.05). Similarly, the plasma IGF-1 concentration was elevated with L-Cit supplementation compared to the CON diet (Figure 2B; *p* < 0.05). The GH concentration in the breast muscle was influenced by the main effects of both environment and diet (*p* < 0.05). It was observed that L-Cit supplementation increased the GH concentration in the breast muscle than the CON diet (Figure 2C; *p* < 0.05). Also, HS promoted the GH concentration in the breast muscle compared to the TNZ condition (*p* < 0.05). However, the IGF-1 concentration in the breast muscle did not differ between all treatment groups (Figure 2D; *p* > 0.05).

### Relative mRNA expression of somatotropic axis-related genes in the breast muscle

**FIGURE 3:**
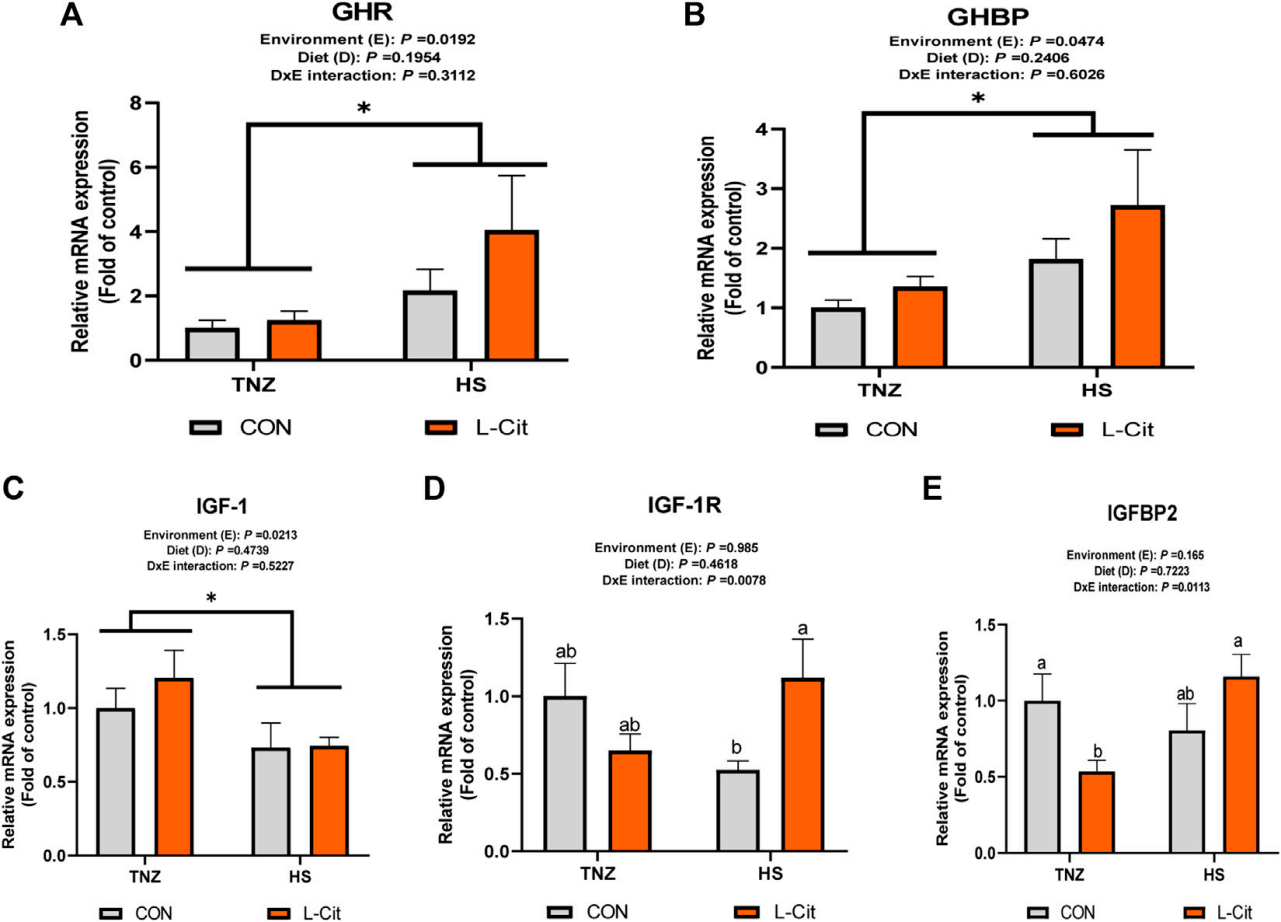
Effect of heat stress and l-citrulline supplementation on somatotropic axis gene in the breast muscle **(A)** Growth hormone receptor (GHR) **(B)** Growth hormone binding protein (GHBP) **(C)** Insulin-like growth factor-1 (IGF-1) **(D)** Insulin-like growth factor-1 receptor (IGF-1R) **(E)** Insulin-like growth factor-1 binding protein 2 (IGFBP2). Data were presented as mean ± SEM (*n* = 8). Means with different alphabetical superscripts indicate significant differences in environment × diet interaction at *p* < 0.05 Asterisk indicates significant main effect of environment at *p < 0.05.

The mRNA expression of GHR and GHBP was significantly induced by HS exposure compared to the TNZ group (Figures 3A,B; *p* < 0.05). In contrast, HS significantly downregulated IGF-1 mRNA expression (Figure 3C; *p* < 0.05). There was a significant effect of environment × diet interaction on the IGF-1R expression in the breast muscle (*p* < 0.05). It was observed that HS + L-Cit upregulated IGF-1R expression higher than the HS + CON group (Figure 3D; *p* < 0.05). In addition, IGFBP2 expression was also upregulated by HS + L-Cit compared to the TNZ + L-Cit group (Figure 3E; *p* < 0.05).

### Protein expression of the mTOR signaling pathway in breast muscle tissues

**FIGURE 4:**
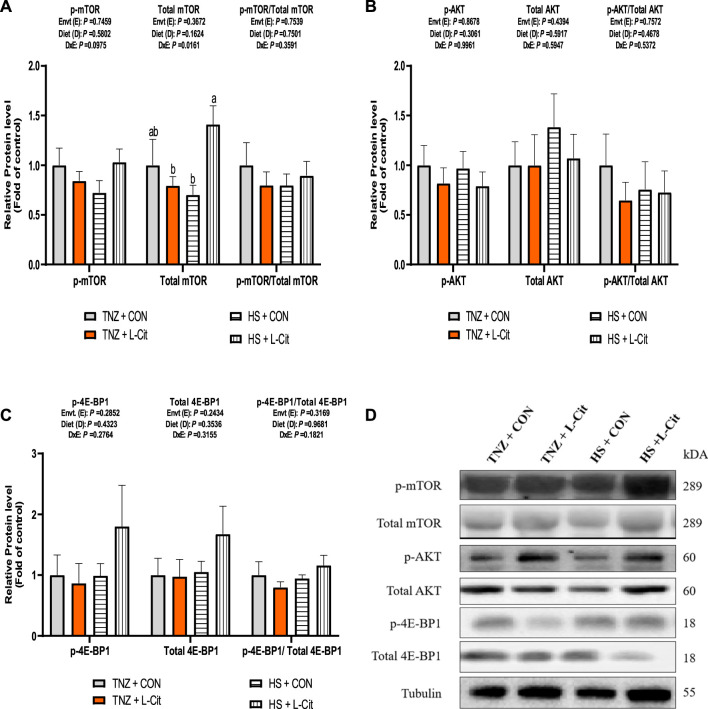
Effect of heat stress and l-citrulline supplementation on mTOR signaling pathway **(A)** phosphorylated, total and ratio of phosphorylated to total mTOR **(B)** phosphorylated, total and ratio of phosphorylated to total AKT **(C)** phosphorylated, total and ratio of phosphorylated to total 4E-BP1 **(D)** Western blot images of tested proteins. Data were presented as mean ± SEM. Means with different alphabetical superscripts indicate significant differences in environment × diet interaction at *p* < 0.05.

The expression of key signaling proteins including mTOR, AKT, and 4E-BP1 in the breast muscle is shown in Figure 4. The p-mTOR expression was not significantly affected by treatments (*p* > 0.05). However, there was a significant effect of environment × diet interaction on the total mTOR expression in the breast muscle of broilers (*p* < 0.05). It was observed that HS + L-CIT increased the total mTOR abundance higher than the HS + CON and TNZ + L-CIT groups (Figure 4B). However, this was not reflected in the p-mTOR/total mTOR ratio. Furthermore, the phosphorylated, total and the ratio of phosphorylated to total protein expressions for AKT and 4E-BP1 in the breast muscle did not differ significantly among the treatments (*p* > 0.05; Figures 4B,C). Figure 4D shows the western blot images of tested proteins between the treatment groups.

## Discussion

Broilers exposed to high ambient temperature exhibit suppressed growth rate, decreased feed consumption, lowered protein retention, and altered protein metabolism (Zuo et al., 2015). In the present study, it was found that HS decreased the body weight, body weight gain, and feed intake of broilers. This corresponds with previous findings that HS impaired the growth performance, carcass traits, and production indices of broiler chickens (Greene et al., 2020; Liu et al., 2020; Ma et al., 2021). Interestingly, L-Cit diet consistently promoted the body weight and body weight gain of broilers. Also, L-Cit supplementation could restore the body weight, body weight gain, and feed intake of HS broilers at certain periods. It was earlier reported that L-Cit supplementation did not influence the growth performance of broilers (Dao et al., 2021), while another study reported that L-Cit showed a tendency to increase the average daily gain in HS pigs (Kvidera et al., 2016). This study demonstrated that L-Cit can stimulate whole body growth in broilers, and to a certain extent, L-Cit can alleviate HS-induced depression on the growth performance of broiler chickens. In line with these findings, L-Cit is known to modulate protein synthesis, muscle growth, and intestinal functions (Chapman et al., 2012; Jegatheesan et al., 2016; Ho et al., 2020). Therefore, these may serve as possible routes for enhanced nutrient absorption and metabolism in HS birds, yielding improvements in the growth performance of broilers.

Broiler chickens exhibit time-dependent physiological reactions when exposed to HS (Zhang et al., 2012). HS exposure increased the circulating concentrations of plasma urea, uric acid, glucose, and total cholesterol. It is understood that the severity and duration of the heat load, species tested and the physiological state of animals can influence glucose clearance and concentration during HS (Xie et al., 2015). In line with our findings, the plasma glucose pool was increased after long-term HS probably to maintain glucose homeostasis during acclimatization to HS condition (Garriga et al., 2006). However, HS decreased the triglyceride contents which may be considered as a metabolic adjustment resulting from lowered feed intake, limitation in hepatic lipid metabolism, or the utilization of triglycerides as an energy source to compensate for reduced energy intake (Sun et al., 2015). Also, the increment in the uric acid (the major end product of protein breakdown in birds) has been attributed to increased protein catabolism (Belhadj Slimen et al., 2016), and amino acid mobilization for gluconeogenesis during HS (Sun et al., 2015; Ma et al., 2018). Therefore, these findings ascertain that HS exposure negatively impacted the nutrient metabolism and physiological responses of broilers.

Exposure to HS decreases the circulating AA flux since the rate of AA inflow (*via* feed intake, muscle synthesis, and AA released from tissues) is lower than the rate of outflow (*via* AA catabolism, AA utilization in protein synthesis, AA losses) (Ma et al., 2021). In this study, the concentration of several essential AA were not affected by HS condition, however, HS exposure diminished the serum concentration of non-essential AA including citrulline, alanine, aspartate, taurine, and 3-methylhistidine. This corroborates with the report that the circulating concentration of several free AA were reduced during long-term HS, whereas, they were increased during short-term HS (Chowdhury et al., 2021). It is known that HS decreases the plasma concentration of certain AA especially those involved in glycogenic pathways due to the increased reliance on hepatic glucose synthesis which is partly achieved through AA catabolism (Baumgard and Rhoads, 2013; Belhadj Slimen et al., 2016). Plasma citrulline was reportedly diminished in HS chicks, and its concentration could serve as HS biomarker in poultry (Chowdhury et al., 2021). This was confirmed in the present study, and we further demonstrated that L-Cit supplementation could restore the serum-free citrulline levels in HS broilers. Unexpectedly, L-Cit supplementation did not influence the plasma levels of key amino acids involved in arginine metabolism such as citrulline, arginine, and ornithine, which differed from the previous report (Uyanga et al., 2020). This disparity is not clearly understood and is subject to further investigations. In addition, L-serine is implicated in various roles related to insulin signaling, glucose homeostasis, mitochondrial function, and neuronal function (Holm and Buschard, 2019). It also participates in various metabolic pathways including mTOR signaling (Zeng et al., 2019). In this study, the serum serine concentration was reduced by L-Cit supplementation under TNZ condition.

L-Cit supplementation was also found to influence the circulating levels of some bioactive peptides such as anserine and carnosine. These peptides are involved in related physiological roles including cellular protection, antioxidation, modulation of muscle contraction, and nutrient metabolism (Wu, 2020). Interestingly, carnosine content was demonstrated to increase in the breast and thigh muscle of stressed birds (Manhiani et al., 2011). Therefore, L-carnosine may function as an anti-heat stressor in poultry (Manhiani et al., 2011; Gao et al., 2020). In the present study, L-Cit decreased the anserine levels during HS, but it exerted differential effects on serum carnosine. L-Cit increased the carnosine concentration under TNZ, whereas, this was diminished under HS condition. The influence of L-Cit on these peptides is not fully ascertained but may be related to their role in muscle physiology since both peptides are involved in skeletal muscle homeostasis (Blancquaert et al., 2016; Suidasari et al., 2016; de Jager et al., 2022). Furthermore, 3-methylhistidine is an amino acid found in actin and myosin, and over 90% of its body pool is present in the skeletal muscle (Williams, 2003). The presence of 3-methylhistidine in the plasma and urine is often considered as a sensitive method for identifying acute changes in the rate of myofibrillar protein degradation. Thus 3-methylhistidine is a useful marker in determining the rate of skeletal muscle degradation (Berridge et al., 2013). In this study, the serum 3-methylhistidine concentration was increased by HS exposure, whereas it was restored to normal levels with L-Cit supplementation to HS broilers. This finding corroborates the report that the plasma 3-methylhistidine level was increased in long-term HS cattle (28°C, 60% RH) (Kamiya et al., 2006). This phenomenon occurs as a result of HS-induced skeletal muscle proteolysis (O’Brien et al., 2010; Tao et al., 2020). Interestingly, L-Cit supplementation to HS broilers diminished the serum 3-methylhistidine levels. This suggests that L-Cit supplementation can alleviate HS-induced protein catabolism and skeletal muscle degradation, validating the role of L-Cit in protein anabolism.

Muscle development is the combined effect of protein synthesis and degradation. The IGF-1/AKT/mTOR pathway is a primary driver and positive regulator in stimulating muscle protein synthesis (Barclay et al., 2019). When the overall rate of protein synthesis exceeds that of protein degradation, muscle hypertrophy occurs (Schiaffino et al., 2013). In broiler chickens, HS adversely affected the growth performance, and protein deposition by altering the transcriptional responses of IGF1, PI3K, and p70S6K in the breast muscle (Zuo et al., 2015). In the present study, HS increased the GH concentration in the breast muscle but did not influence IGF-1 concentration in the plasma and breast muscle. Rather, the responsiveness of the GH/IGF signaling to HS was increasingly evident at the transcriptional level. Within the breast muscle, HS upregulated GHR, and GHBP expression but downregulated IGF-1 and IGF-1R expression. Therefore, HS induction of GH concentration and GHR expression, with its subsequent downregulation of IGF-1 expression in the breast muscle may be indicative of alterations in protein metabolism. In line with this, it is known that HS promotes protein catabolism and muscle breakdown to drive the gluconeogenic pathway (Ma et al., 2021).

In addition, the hypothalamic gene expression revealed that HS decreased the GHR, GHBP, and IGF-1 mRNA expression levels. As such, the downregulated IGF-1 expression may be caused by deficits in IGF-1R signaling in HS animals. In line with this, it was reported that the plasma IGF-1 was initially increased in HS cows, but it tended to decline with increasing thermal load (Rhoads et al., 2009). In another study, HS reduced the intracellular GH signaling *via* a synchronous reduction in the relative abundance of hepatic GHR, GHR1A, and IGF-1 genes, suggesting that HS uncoupled the GH-IGF axis (Rhoads et al., 2010). In a study conducted to understand the metabolic variations in chronic HS broilers, it was found that a significant amount of the metabolites that were modulated in the muscle and plasma were related to protein and energy metabolism (Zampiga et al., 2021). Therefore, since the skeletal muscle is a highly metabolic tissue involved in protein and energy homeostasis, this may account for the upregulated expression of these growth factors in the breast muscle during HS rather than their downregulated expression in the hypothalamus of broilers. To corroborate our findings, it was demonstrated that chronic HS downregulated the gene expressions of IGF-1, IGF-1R, and mTOR in the breast muscle of broilers (Ma et al., 2018). Therefore, this study revealed tissue-specific responses of the GH/IGF-1 signaling during HS conditions, and that HS altered protein synthesis by dysregulating the GH/IGF-1 axis. An underlying limitation of the current study was that the muscle yield was not computed, however, since the skeletal muscle proportion accounts for about 45 percent of broiler’s carcass weight (Kokoszyński and Bernacki, 2008), it is ascertained that HS reduction of whole body weight is highly associated with muscle weight loss in broilers.

Furthermore, this study demonstrated that L-Cit supplementation increased the circulating GH and IGF-1 levels, as well as the GH concentration in the breast muscle. This may account for the improved growth response observed in L-Cit fed broilers, arising from increased peripheral GH and IGF-1 transport to target tissues. Also, the hypothalamic GHBP expression, as well as IGF-1R and IGFBP2 expression in the breast muscle were upregulated by L-Cit under HS condition. Several studies have reported the ability of citrulline to positively influence muscle growth and function in rodent and human models. In old malnourished rats, citrulline supplementation increased the rate of protein synthesis rate and muscle protein content (Osowska et al., 2006). Refeeding citrulline-supplemented diet (5 g Cit/kg/day) to malnourished rats significantly improved the muscle mass and motor activity (Faure et al., 2012). Treatment of L-Cit alone stimulated the muscle protein synthesis and increased muscle strength during diet restriction in rats (Ventura et al., 2013). *In vitro* test using cultured C2C12 myotubes showed that L-Cit improved the protein synthesis rate, myotube diameter, and protected muscle myotubes from cachectic stimuli (Ham et al., 2015). All these reports validate that L-Cit plays an important role in promoting skeletal muscle functions under stress conditions. In the present study, it was found that L-Cit exerted protective effects on the growth responses of HS broiler. Alongside this, L-Cit had modulatory effects on the circulating levels of the GH/IGF-1 hormones and the gene expression of some growth factors in the hypothalamus and breast muscle of HS broilers.

The mTOR signaling pathway in poultry and mammals is crucial for regulating protein synthesis (Zuo et al., 2015). More so, mTOR phosphorylation at Ser2448 is a key signal for cell growth (Reiling and Sabatini, 2006). In the present study, L-Cit supplementation increased the protein abundance of total mTOR in the breast muscle of HS broilers. Unexpectedly, the changes in the total mTOR were not associated with any variations in the phosphorylation and total protein expression of mTOR’s upstream and downstream targets (that is, AKT and 4E-BP1). It was previously reported that within the soleus and plantaris muscles of Wistar rats exposed to thermal stress, the phosphorylation of AKT and p70S6K were significantly induced, whereas that of mTOR and 4E-BP1 phosphorylation were unaffected and the total protein expression levels of AKT, mTOR, p70S6K, and 4E-BP1 were unchanged during HS exposure (Yoshihara et al., 2013). This suggests that HS may influence the mTOR signaling in a temperature-dependent manner. Additionally, citrulline can regulate muscle protein synthesis *via* the mTORC1 signaling pathway and activation of its downstream effectors, S6K1 and 4E-BP1 (Le Plenier et al., 2012; Goron et al., 2017; Le Plénier et al., 2017; Breuillard et al., 2019; Filippi et al., 2021). Therefore, the lack of significant changes in mTOR’s upstream and downstream targets suggests other possible routes for its activation since it is a key metabolic sensor involved in multiple biological activities.

**FIGURE 5:**
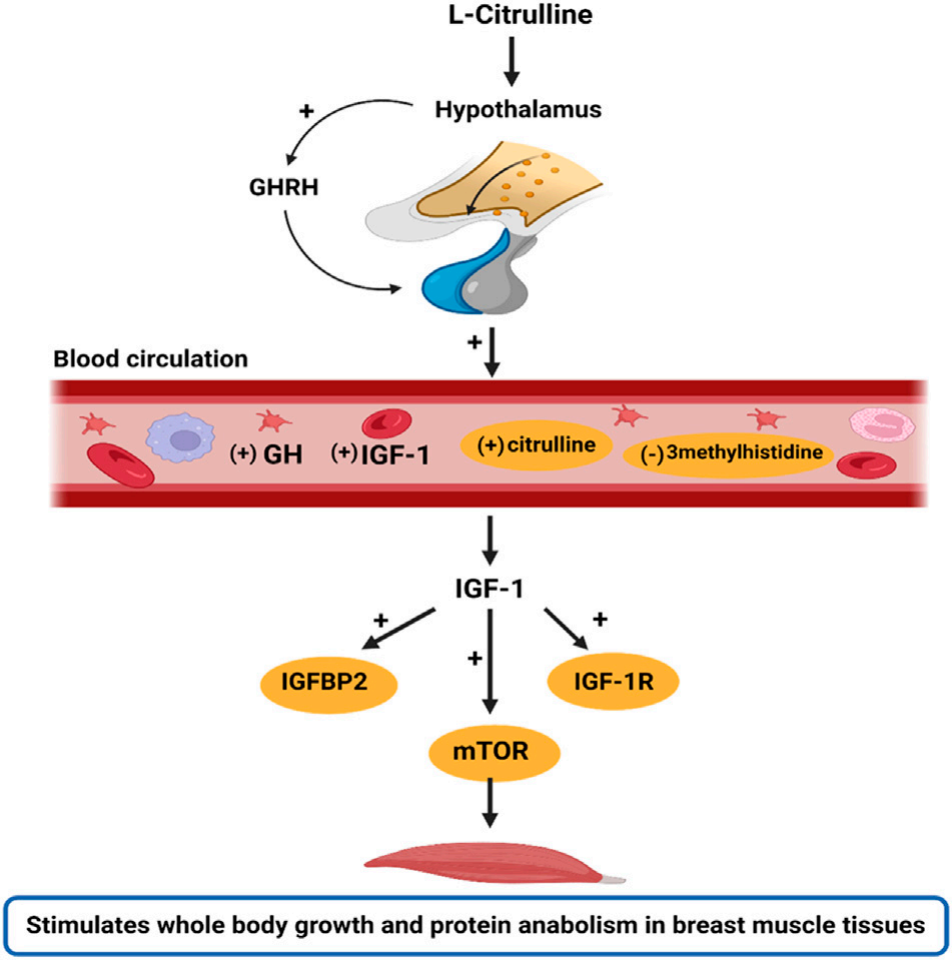
Schematic illustration of the stimulatory effects of l-Citrulline on the GH/IGF-1 axis. The GH/IGF-1 axis is important in regulating body growth. In this study, it was found that with l-Citrulline supplementation, there was a stimulatory release of GHRH from the hypothalamus. The GHRH is responsible for GH production and secretion by the somatotrophs in the anterior pituitary gland. The GH released binds to GH receptors and induces IGF-1 production. This was evidenced by the increased peripheral circulation of GH and IGF-1 in the blood circulation with l-Citrulline supplementation. In the circulation, IGF-1 bioavailability allows for its activation of various pathways, including the mTOR signaling that promotes cell growth, metabolism, and protein synthesis. Thus, we hypothesize that l-Citrulline acts via its stimulation of GHRH to activate the GH/IGF-1 axis. Interestingly, under heat stress exposure, l-Citrulline can activate the mTOR signaling to maintain body growth and limit protein catabolism. These actions were also evidenced by l-Citrulline’s regulation of certain amino acids during heat stress such as its induction of circulating citrulline and diminishing of 3methylhistidine, a marker of muscle protein degradation. Note: (+) indicates stimulatory response; (−) indicates inhibitory response; yellow circling indicates the actions of l-citrulline during heat exposure. The image was created with Biorender.

Noteworthy, this study did not incorporate an isonitrogenous control in the diet formulation. However, since L-Cit is not a constituent of most proteins sources, it is not among the primary amino acids encoded by the DNA, nor does it participate in protein synthesis (Curis et al., 2005; Mandel et al., 2005), it is suggested that the inferences drawn from L-Cit in the present study are attributed to the direct effects of supplemental L-Cit provided in the trial. Altogether, these findings reveal that HS depresses the growth performance of broilers, and dysregulates GH/IGF signaling. However, L-Cit supplementation could regulate the GH/ IGF-1 axis to stimulate protein anabolism and promote whole-body growth (Figure 5). Also, L-Cit supplementation exerted protective effects on the growth performance, amino acid homeostasis, gene expression of growth factors, and mTOR abundance in HS broilers.

## Data Availability

The datasets presented in this study can be found in online repositories. The names of the repository/repositories and accession number(s) can be found below: https://www.ncbi.nlm.nih.gov/genbank/, XM_015296360;https://www.ncbi.nlm.nih.gov/genbank/, M74057.1;https://www.ncbi.nlm.nih.gov/genbank/, DQ138367.1;https://www.ncbi.nlm.nih.gov/genbank/, NM_001004384.2;https://www.ncbi.nlm.nih.gov/genbank/, AF041800.1;https://www.ncbi.nlm.nih.gov/genbank/, NM_205359.1;https://www.ncbi.nlm.nih.gov/genbank/, NM_205518.

## Abbreviations

4E-BP1, Eukaryotic translation initiation factor 4E-binding protein one; AA, Amino acids; AKT, Protein kinase B; ANOVA, Analysis of variance; BCA, Bicinchoninic Acid; cDNA, Complementary deoxyribonucleic acid; CON, Basal diet; ECL, Enhanced chemiluminescence; GH, Growth hormone; GHBP, Growth hormone binding protein; GHR, Growth hormone receptor; GHR1A, Growth hormone-receptor 1A; GHRH, Growth hormone releasing hormone; HRP, Horseradish peroxidase; HS, Heat stress; IGF-1, Insulin-like growth factor-1; IGF-1R, Insulin-like growth factor-1 receptor; IGFBP2, Insulin-like growth factor-1 binding proteins two; IgG, Immunoglobulin G; L-Cit, l-Citrulline; mTOR, Mammalian target of rapamycin; mTORC1, Mammalian target of rapamycin complex one; p70S6K, 70 kDa ribosomal S6 kinase; PI3K, Phosphatidylinositol 3′-kinase; PVDF, Polyvinylidene fluoride; RH, Relative humidity; RIPA, Radioimmunoprecipitation assay; RNA, Ribonucleic acid; S6K1, Ribosomal protein S6 kinase beta-1; SDS-PAGE, Sodium Dodecyl Sulfate-Polyacrylamide Gel Electrophoresis; TMB, 3,3′, 5,5″-tetramethylbenzidine; TNZ, Thermoneutral.
